# Platelet-Derived Growth Factor Stimulated Migration of Bone Marrow Mesenchymal Stem Cells into an Injectable Gelatin-Hydroxyphenyl Propionic Acid Matrix

**DOI:** 10.3390/biomedicines9020203

**Published:** 2021-02-17

**Authors:** Wanting Niu, Teck Chuan Lim, Abdulmonem Alshihri, Ravikumar Rajappa, Lishan Wang, Motoichi Kurisawa, Myron Spector

**Affiliations:** 1 Tissue Engineering and Regenerative Medicine Laboratories, VA Boston Healthcare System, Boston, MA 02130, USA; wniu@bwh.harvard.edu (W.N.); teckchuan@gmail.com (T.C.L.); monem.alshihri@post.harvard.edu (A.A.); cra.dr.ravikumar@gmail.com (R.R.); 2 Department of Orthopedic Surgery, Brigham and Women’s Hospital, Harvard Medical School, Boston, MA 02155, USA; 3 Harvard-MIT Division of Health Sciences and Technology, Cambridge, MA 02139, USA; 4 Department of Restorative Dentistry and Biomaterials Sciences, Harvard School of Dental Medicine, Boston, MA 02155, USA; 5 College of Dentistry, King Saud University, Riyadh 12372, Saudi Arabia; 6 Institute of Bioengineering and Nanotechnology, Singapore 138669, Singapore; lishanwang@gmail.com (L.W.); mkurisawa@ibn.a-star.edu.sg (M.K.)

**Keywords:** bone marrow mesenchymal stem cells, migration, proliferation, platelet-derived growth factor, stromal cell-derived factor-1α

## Abstract

Bone marrow mesenchymal stem cells (bMSCs) are responsible in the repair of injured tissue through differentiation into multiple cell types and secretion of paracrine factors, and thus have a broad application profile in tissue engineering/regenerative medicine, especially for the musculoskeletal system. The lesion due to injury or disease may be a closed irregular-shaped cavity deep within tissue necessitating an injectable biomaterial permissive of host (endogenous) cell migration, proliferation and differentiation. Gelatin-hydroxyphenyl propionic acid (Gtn-HPA) is a natural biopolymer hydrogel which is covalently cross-linked by horseradish peroxidase (HRP) and hydrogen peroxide (H_2_O_2_) in situ and can be delivered to the lesion by needle injection. Growth factors and cytokines can be directly incorporated into the gel or into nano- and micro-particles, which can be employed for sustained release of biomolecules while maintaining their bioactivity. In this study, we selected polyelectrolyte complex nanoparticles (PCNs) prepared with dextran sulfate and chitosan as the carrier for platelet-derived growth factor (PDGF)-BB and stromal cell-derived factor (SDF)-1α, which have been tested effectively in recruiting stem cells. Our in vitro results showed a high degree of viability of bMSCs through the process of Gtn-HPA covalent cross-linking gelation. The Gtn-HPA matrix was highly permissive of bMSC migration, proliferation, and differentiation. PDGF-BB (20 ng/mL) directly incorporated into the gel and, alternatively, released from PCNs stimulated bMSC migration and proliferation. There were only small differences in the results for the direct incorporation of PDGF into the gel compared with its release from PCNs, and for increased doses of the growth factor (200 ng/mL and 2 µg/mL). In contrast, SDF-1α elicited an increase in migration and proliferation only when released from PCNs; its effect on migration was notably less than PDGF-BB. The in vitro results demonstrate that PDGF-BB substantially increases migration of bMSCs into Gtn-HPA and their proliferation in the gel, and that these benefits can be derived from incorporation of a relatively low dose of the growth factor directly into the gel. These findings commend the use of Gtn-HPA/PDGF-BB as an injectable therapeutic agent to treat defects in musculoskeletal tissues.

## 1. Introduction

For certain regenerative medicine applications, the reparative potential of endogenous stem and progenitor cells including mesenchymal stem cells (MSCs) may be able to be tapped by recruitment of the cells into the injury site. Two of the issues that need to be addressed for applications in which a cavitary defect or otherwise stroma-less lesion has resulted from injury or disease are: the introduction of a defect-filling matrix to accommodate the migration of reparative cells into the lesion; and a chemoattractant for the target cells to recruit adequate numbers into the matrix. Irregularly shaped lesions of less than approximately 1 cm^3^, which are contained in healthy tissue, may necessitate matrices that can be injected in order to limit the secondary damage resulting from surgical implantation of a pre-formed matrix.

In recent years, several types of injectable polymers have undergone investigation, including: photo-polymerizable polymers [1]; self-assembling peptides [2]; shear-thinning (thixotropic) polymers [3]; and cryo gels [4,5]. Another class of injectable polymers is capable of undergoing enzyme-mediated covalent cross-linking (e.g., horseradish peroxidase, tyrosinase, laccase, transglutaminase) In the horseradish peroxidase (HRP)-induced polymerization, the enzyme catalyzes the di-phenolic formation of phenol-conjugated polymer backbones (e.g., gelatin, hyaluronic acid) with hydrogen peroxide (H_2_O_2_) [6,7,8,9]. One of these polymers, gelatin-hydroxyphenyl propionic acid (Gtn-HPA), which is a natural biopolymer-based hydrogel that is covalently cross-linkable in vivo through the oxidative coupling of phenol moiety catalyzed by HRP and propagated by H_2_O_2_, Reference [9] has been shown to accommodate neural stem cell migration, Reference [10] astrocyte activation and proliferation [11], recruit bMSC migration and osteogenic differentiation [12], and promote MSC endothelial differentiation and tube formation under certain conditions [13,14].

The cytokine, stromal cell-derived factor (SDF)-1α [15,16], and growth factor, platelet-derived growth factor (PDGF)-BB [17], which are upregulated in injured tissues, have been shown to be chemoattractant and mitogenic, respectively, for MSCs. SDF-1α is secreted by the activated monocytes or macrophages during the acute inflammation period, which serves as an MSC recruiter in bone regeneration [18]. PDGF has been approved by the Food and Drug Administration (FDA) for wound healing applications [19]. Prior studies [20,21] have demonstrated that PDGF-BB can stimulate the migration of rat or human mesenchymal bone marrow cells, using a Boyden chamber, compared to a host of other growth factors, including BMP-2, -4, and -7, TGF-β, FGF-2, and IGF-1. Additionally noteworthy, is the effect of PDGF-BB in regulating the behavior of blood vessel pericytes [22], which have been tied to the origin of MSCs [23] in several tissues including bone marrow [24].

Implementation of chemoattracting factors such as SDF-1α and PDGF-BB for the recruitment of endogenous MSCs into a biomaterial matrix-filled defect can be by the simple addition of the protein into the matrix or by incorporation of the growth factor into a secondary delivery vehicle such as a nanoparticle; nanoparticles offer the potential for a greater degree of control over the sustained release of the proteins. Polyelectrolyte complex nanoparticles (PCNs), one type of nanoparticle that has been employed for protein release, is prepared from dextran sulfate (DS) and chitosan (CS) from aqueous phase [25,26]. PCNs have high protein encapsulation efficiency (~85%) and rapid initial release properties and may physically protect the proteins/peptides from proteinase cleavage, to serve as controlled release vehicles for growth factors.

The aim of our study was to evaluate the effects of PDGF-BB and SDF-1α on bMSC migration and proliferation in Gtn-HPA. PDGF-BB and SDF-1α were incorporated either directly into the Gtn-HPA or via PCNs. We hypothesized that by supporting the migrating cells with a biocompatible matrix and by attracting them with sustained release of a growth factor or cytokine, the Gtn-HPA gel can successfully recruit and mobilize bMSCs. Those factors [12] that also work as mitogens could furthermore stimulate the proliferation of the recruited cells at the site.

## 2. Experimental Section

### 2.1. Synthesis and Gelation of Injectable Gelatin-Hydroxyphenyl Propionic Acid Matrix

Gtn-HPA was synthesized from gelatin (Wako Pure Chemicals Industries, Osaka, Japan) and HPA (Sigma-Aldrich, St. Louis, MO, USA) as reported [9]. In this study, 2% Gtn-HPA dissolved in a solution comprising 50% Dulbecco’s phosphate-buffered saline (DPBS, Invitrogen, Carlsbad, CA, USA) and 50% Dulbecco’s Modified Eagle Medium-low glucose (DMEM-LG; Invitrogen) was cross-linked with 0.1 U/mL HRP; (Wako Chemicals, USA) and 1.2 mM H_2_O_2_ (Sigma-Aldrich, USA). Gelation was completed by 1 h at 37 °C. For experimental groups with Gtn-HPA matrices containing PCNs, protein-encapsulated PCNs or PCNs without protein (“blank” PCNs) were mixed homogenously with the Gtn-HPA polymer solution before gelation, to a final protein concentration of 20 ng/mL, 200 ng/mL or 2 µg/mL.

The chemical structure, preparation and characterization of the gels have previously been published [9].

### 2.2. Synthesis and Evaluation of Protein-Encapsulated Dextran/Chitosan Polyelectrolyte Nanoparticles

PDGF-BB encapsulated PCNs (PDGF-BB/PCNs) were made by combining dextran sulfate (DS), chitosan (CS), and PDGF-BB at a ratio of 1:0.33:0.2 [18]. Briefly, we mixed 0.5 mg/mL recombinant human PDGF-BB (Peprotech, Rocky Hill, NJ, USA) in 5% D-Trehalose dihydrate, (Sigma, USA) and 0.25% DS (MW 500 kDa; Fisher Scientific, Waltham, MA, USA). After 30 min stirring, 0.1% CS (in 0.175% acetic acid, pH 5.5, MW 10 kDa; Polysciences, Warrington, PA, USA) was added into the solution dropwise and stirred for a further 30 min. Ultimately, 15% mannitol (Sigma, USA) was dropped in and stirred for another 10 min. The PCNs were harvested by centrifuging at 15,000× *g* for 20 min at 4 °C and resuspended with 5% mannitol twice before lyophilizing overnight. SDF-1α encapsulated PCNs (SDF-1α/PCNs) and blank PCNs were prepared analogously, but with/without 0.5 mg/mL recombinant rat SDF-1α (Peprotech, USA), respectively. Protein encapsulated PCNs was resuspended with dI H_2_O to 0.2 mg/mL protein before adding into gel solution. Blank PCNs (control) concentration was adjusted to the same of (DS+ CS) concentration in protein encapsulated PCNs.

Supernatants removed from the centrifuge step were collected in protein low bind tubes. Proteins and DS in the supernatants not encapsulated into PCNs were detected with BCA protein assay (Fisher Scientific, USA) and toluidine blue assay (EMD Millipore, Burlington, MA, USA), respectively. The PCNs mass was estimated as the weight of encapsulated protein and DS and the input mass of CS (supposing CS was encapsulated totally). Protein encapsulation efficiency was calculated as: (Input mass of protein- BCA detected protein)/Input mass of protein ×100%. Usually, the encapsulation efficiency could be over 80%.

Properties of the PCNs have previously been reported: cytocompatibility of the PCNs, SDF-1α release kinetics, mean particle size and polydispersity index [18]. The physical characterization and transmission electron microscopy of comparable PCNs has also been reported by Ramasamy, et al. [27].

### 2.3. In Vitro Protein Release from the Polyelectrolyte Complex Nanoparticles

PDGF-BB/PCNs and SDF-1α/PCNs were prepared by suspending the agents in a release buffer—1 mM PBS (pH 7.4) with 0.02% NaN_3_—for a final concentration of 50 µg/mL protein. The PCNs incorporating the proteins were aliquoted into 6 sterile low protein binding tubes, with 1 mL in each tube, and then the tubes were shaken at 100 times/min at 37 °C. For the first seven days, supernatants were collected every day, and, thereafter, every other day until the 28th day, by centrifuging at 4 °C, 15,000× *g* for 20 min. The same volume of release buffer was added back to resuspend the protein-encapsulating PCNs. The amount of protein was quantified by BCA assay.

### 2.4. Mesenchymal Stem Cell Culture and Viability of the Cells in Gelatin-Hydroxyphenyl Propionic Acid Matrix

The aspiration and sub-culture of bone marrow stem cells was approved by our institutional animal care and use committee (IACUC). bMSCs were obtained from heparinized bone marrow from the iliac crests of an adult Spanish goat when it was sacrificed for other purposes [28,29] and identified as stem cells, as previously reported in our former publications [30]. As the cells from various goats behaved in comparable fashion, in the current study, in order to remove any interindividual variability (viz., age), cells from only one goat were employed. The stemness of the cells were also identified by immunofluorescence (IF) staining of the surface markers, CD 29, CD 44, CD73 (negative, not shown), CD 90 (negative, not shown) and CD 105. Cells were expanded in monolayer within MSC expansion medium: DMEM-LG containing 10% fetal bovine serum (FBS; Invitrogen); 100 units/mL penicillin and 100 µg/mL streptomycin (Invitrogen); and 10 ng/mL recombinant human fibroblast growth factor-2 (FGF-2; R&D Systems, USA). Our prior studies also found the MSCs retained their stemness through 2 sub-cultures, in order to get enough cells, all cells were used at passage 2, when they reached 80% confluence, with the density of 1 × 10^5^ cells/mL in gel solution. Expansion medium without FGF-2 was added on top of the gels when the gelation was completed, then the medium was changed bi-daily.

A volume of 100 µL of 2% Gtn-HPA gel containing 10^4^ bMSCs was spread in the center of a 35-mm diameter cell culture dish (*n* = 6) to form a round, thin layer about 1 cm in diameter. After 20 min incubation at 37 °C, 3 mL of expansion medium was added to the dish. Cells were stained, after a 24-h incubation period, with the Live/Dead viability/cytotoxicity kit (Invitrogen) using calcein acetoxymethyl ester (Calcein AM) and ethidium homodimer-1 (EthD-1), for 45 min at 37 °C. The staining reagent containing medium was replaced by DPBS for 30 min to further remove the extra staining dyes. Eventually, the cell-containing gels were imaged under a fluorescent microscope. Dead and live cells were counted in 10 randomly selected images per gel.

### 2.5. Annulus/Core Migration Model

A 3-dimensional migration assay (*n* = 8) was constructed using an annulus/core model [16]. The core Gtn-HPA matrix was made by casting 236 µL 2 wt.% Gtn-HPA, cross-linked by 0.1 U/mL HRP and 1.2 mM H_2_O_2_, into a mold with a diameter of 8.7 mm. After 1 h incubation at 37 °C, the cylinder of Gtn-HPA was removed from the mold and transferred to the center of a well of a 24-well plate. The core Gtn-HPA matrix incorporated one of the following: nothing (i.e., blank control gel); blank PCNs; 20 ng/mL, 200 ng/mL, or 2 µg/mL PDGF-BB or 2 µg/mL SDF-1α; or PCNs containing the same amount of the proteins. The annulus cell-containing gel, which was used as the tissue simulant, was made up of 10^5^ bMSCs/mL in 2 mg/mL rat tail type I collagen (BD Biosciences, Franklin Lakes, NJ, USA), incorporating 10 ng/mL FGF-2 (Figure 5A).

The number of bMSCs migrating into the core gel, and the distance they penetrated into the core gel were measured in micrographs (Olympus IX51, Tokyo, Japan) taken on the 4th and 7th day, using 4 typical fields of view for each sample. Data were normalized as cell number/mm of perimeter for statistical analysis. Migration distance was measured from each cell body to the interface on day 7 images, using Image Pro Plus 6.0 (Mayer Instruments, Houston, TX, USA).

### 2.6. Bilayer Proliferation Model

A volume of 200 µL Gtn-HPA gel (including the same experimental and control groups; *n* = 6) was cast on the bottom of a 48-well plate and incubated for 1 h at 37 °C. Then, 100 µL of 2 mg/mL type I soluble collagen gel seeded with 10^5^ cells/mL bMSCs was cast on top of the Gtn-HPA base (Figure 5A) and incubated at 37 °C for 30 min to gel the collagen before adding 200 µL FGF-2-free expansion medium.

On days 0, 4 and 7, the cultures were terminated, and the medium was replaced by 500 µL of 1000 U/mL collagenase I solution to digest the gel system and free the bMSCs. After 30 min of enzymatic degradation, cells were collected by 3500 rpm (1300 *g*) × 5 min centrifugation. The number of cells was measured by the PicoGreen DNA quantification assay (Invitrogen Molecular Probes, Carlsbad, CA, USA).

### 2.7. Immunofluorescence Staining

bMSCs were seeded in a 6-well cell culture plate with a density of 5000 cells/cm^2^ and cultured for 24 h. The cells were fixed with 4% paraformaldehyde (PFA) at 4 °C overnight then permeabilized by 0.1% Triton X-100 (Fluka Analytical, Buchs, Switzerland). The cells were stained with anti-CXCR4 antibody (Abcam, Cambridge, MA, USA, ab2074, 1:100 dilution), anti-CD 29 (Novus Biologicals, Englewood, IL, USA, NBP1-97671, 1:100 dilution), anti-CD 44 antibody (Novus Biologicals, NBP2-22530, 1:100 dilution), anti-CD73 (Santa Cruz Biotechnology, sc-25603, 1:100 dilution), anti-CD90 (Abcam, ab225, 1:100 dilution), and anti-CD105 antibody (Abcam, ab156756, 1:100 dilution) at room temperature for 4 h, followed by secondary Alexa 488 donkey anti-rabbit IgG (5 µg/mL, Jackson Immuno Research, West Grove, PA, USA) at room temperature for 1 h, and 4′6-diamidino-2-phenylindole dihydrochloride (DAPI, 0.2 µg/mL, Invitrogen, Carlsbad, CA, USA) to counterstain nuclei for 5 min. CXCR4 expression was detected by using a fluorescent microscope (Olympus BX60, Tokyo, Japan) with a 10 ×-objective. The stemness markers were imaged by a confocal microscopy (Nikon C2, Tokyo, Japan) with a 10 ×-objective.

### 2.8. Quantification of Auto-Osteogenic Differentiation in Gtn-HPA Gels

To investigate if the migrated bMSCs maintained their stemness in the Gtn-HPA gels, inductively coupled plasma atomic emission spectroscopy (ICP-AES, HORIBA Jobin Yvon, Edison, NJ, USA) was used to quantify calcium deposited in gel by bMSCs as they underwent osteogenic differentiation. bMSCs were embedded in 2% Gtn-HPA gels at 10^5^ cell/mL. The groups included acell-free gel control, and cell-seeded gels maintained in expansion medium or in osteogenic medium. A volume of 1 mL gel solution was cast into each well of a 24-well plate (*n* = 4) and incubated at 37 °C for 30 min before adding 0.5 mL of expansion medium into each well. On the second day, for the osteogenic group, the medium was changed to osteogenic medium supplemented with 100 nM dexamethasone, 10 mM β-glycerol phosphate and 0.05 mM ascorbic acid in expansion medium; the medium in the expansion medium group was also changed simultaneously. The media were changed every 2 days until 4 weeks, and the gels were then fixed with PFA.

Gel specimens were carefully removed from the 24-well plates and rinsed with dI H_2_O several times, 5 min each time until the gel was transparent, to completely remove the medium (which contained calcium) and PFA in the gels. The gels were dissolved in 250 µL of ultrapure nitric acid (Omni Trace, 69–70%, EMD Millipore, USA) for 24 h on a shaker at 37 °C until no visible remnants were visible. The gel solution was diluted with high purity dI H_2_O (reagent grade, Ricca chemical company, Arlington, TX, USA) to 10 mL with the final concentration of HNO_3_ was 2% (*w*/*w*). The gel solution was filtered through a 0.2 µm filter (Millipore, USA) before pumping into the ICP machine. Calibration standards were diluted from 1000 µg/mL calcium ICP standard (ULTRAgrade Solution, Ultra Scientific, Santa Clara, CA, USA) to series of concentration at 0, 0.2, 0.5, 2, 10 and 20 µg/mL with the same 2% HNO_3_ used above. The Ca values were measured at the wavelength λ = 317.933 nm 3 times for each sample.

### 2.9. Statistical Analysis

Statistical Analysis was performed using STATVIEW software. Values are expressed as means ± standard errors. Statistical significance was determined by one-factor analysis of variance (ANOVA) followed by Fisher’s protected least square difference (PLSD) post hoc test, with a significance criterion of *p* < 0.05.

## 3. Results

### 3.1. bMSC Phenotype and Viability in Gtn-HPA Matrices

**Figure 1:**
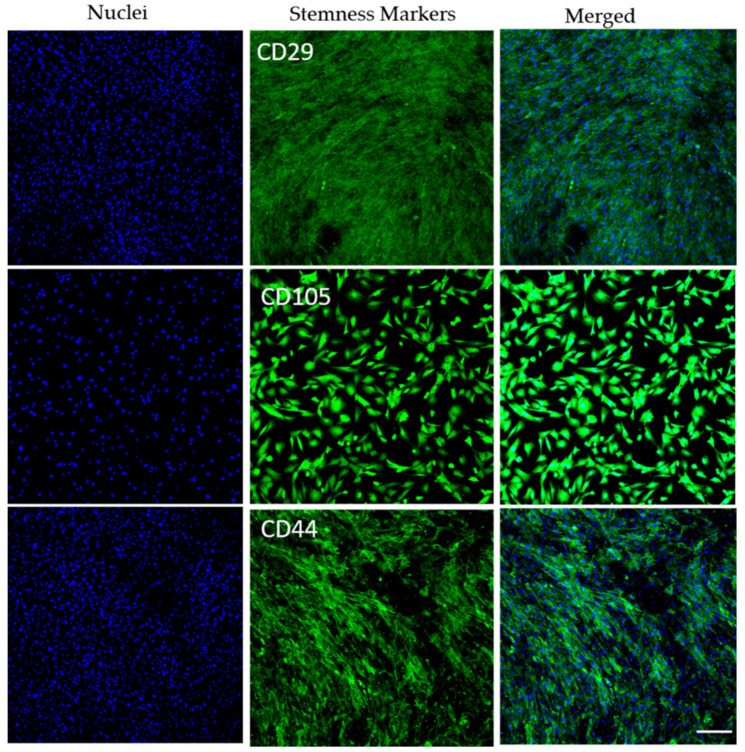
The surface stemness markers of the goat bone marrow mesenchymal stem cells. The CD 29 and CD 44 were found moderate positive, and CD 105 was stained as highly positive. However, CD 73 and CD 90 were identified as negative under the same imaging conditions. (scale bar = 200 µm).

Prior studies from our laboratory employing caprine bMSCs similarly isolated from aspirated caprine marrow demonstrated their stemness in chondrogenic assays [30,31]. In this study, we further identified the surface stemness markers of these bMSCs, and found the CD105, an accessory receptor for transforming growth factor (TGF)-β, is highly positive; whereas CD 44 and CD29 had moderate positive signals, but CD 90 and CD73 were found negative when they were imaged under the same conditions (Figure 1). The negative staining of CD 73 and CD90 agrees with a prior study by Rozemuller et al. [32]. However, in their study, the staining of CD105 was also found negative, which may due to the lack of reactivity of the selected antibody with the goat.

**Figure 2:**
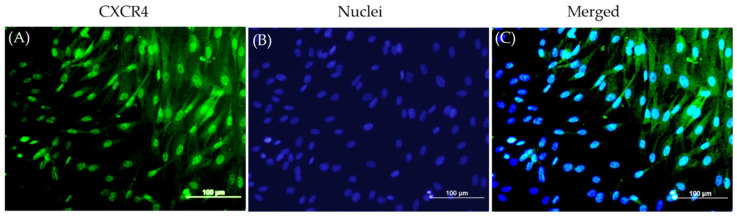
CXCR4 expression on bMSCs (**A**) bMSCs in monolayer stained by immunofluorescence with an anti-CXCR4 antibody (green). (**B**)Nuclei counter stained by DAPI (blue). (**C**) Merged CXCR4 and nuclei staining. Scale bars are 100 µm.

Other studies in the literature have established PDGF-BB and SDF-1α [33,34] as chemoattractants for bMSCs; our immunofluorescence staining of the bMSCs in monolayer revealed the goat bMSCs that expressed CXCR4, the main receptor for SDF-1α, on their plasma membrane (Figure 2).

**Figure 3:**
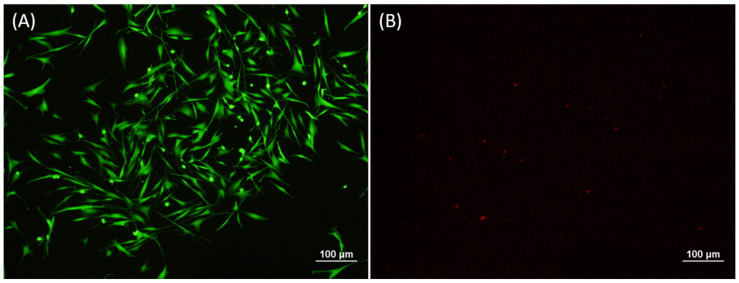
Cell viability after 24 h culture in Gtn-HPA matrix. (**A**) Live cells stained by Calcium AM. (**B**) Dead cells stained by EthD-1. Scale bars are 100 µm.

In assessing the effects of the HRP- and H_2_O_2_-mediated covalent cross-linking process on the bMSCs, we found the large majority of bMSCs (99 ± 0.005%; *n* = 3; mean ± standard error of the mean) exposed to the Gtn-HPA cross-linking process using 0.1 U/mL HRP and 1.2 mM H_2_O_2_ and grown in the matrix in expansion medium for 24 h remained viable, as reflected by their green stain by Calcein-AM; few cells stained red with EthD-1, indicative of dead cells. After 24 h, most cells displayed a spread morphology typical of MSCs in monolayer or a spindle-shaped appearance that may have been partly due to the examination of the spread cells on edge. A fraction of the cells appeared to be rounded (Figure 3).

### 3.2. Release Rate of PDGF-BB and SDF-1α from Polyelectrolyte Nanoparticles

**Figure 4:**
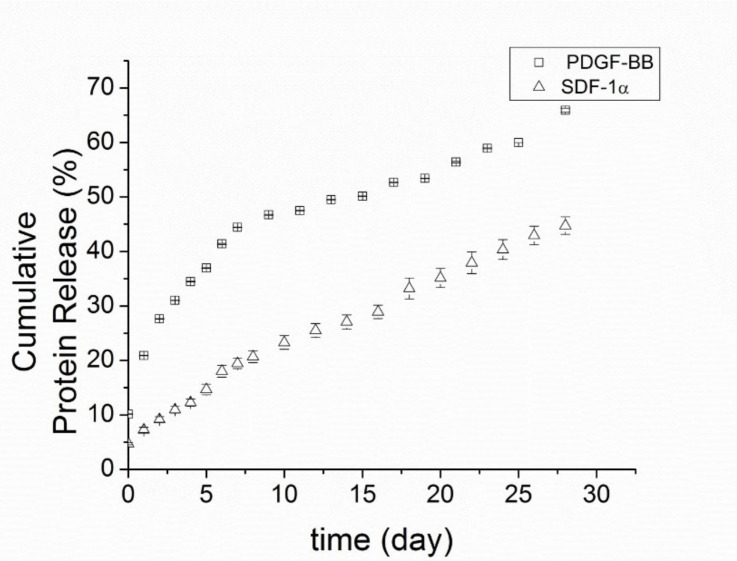
PDGF-BB and SDF-1α release profile from 2% Gtn-HPA gel.

For PDGF-BB/PCNs, there was a burst release of 10 ± 0.05% of the total protein immediately into the buffer soon after adding the release buffer, followed by another 11 ± 0.06% of the protein released in the first 24 h (Figure 4). Thereafter, the release rate slowed over the second day, maintaining a stable release rate of around 2.5–4.5% of total protein per day for the following10 days. In the ensuing 18 days, the release rate slowed to 0.75–3% of total protein per day. In total, (65.9 ± 1.4) % of the overall amount of protein was released in 28 days.

Because SDF-1α is more positively charged than PDGF-BB, with a higher isoelectric point (pI = 9.92 compared to pI = 9.8), SDF-1α bound more avidly to dextran sulfate at pH 7.4. Therefore, SDF-1α/PCNs yielded a slower release rate and a longer sustained release time than PDGF-BB. There was only a modest burst release of protein (~4.7 ± 0.2%) from SDF-1α/PCNs after the addition of the release buffer, and only around 7.3 ± 0.7% of total SDF-1α was released from the vehicles in the first 24 h. The releasing profile of SDF-1α/PCNs was gradual, with about 45 ± 2% of the total SDF-1α released in 28 days.

### 3.3. Migration of bMSCs into the Gtn-HPA Matrix under the Influence of PDGF-BB and SDF-1α

**Figure 5:**
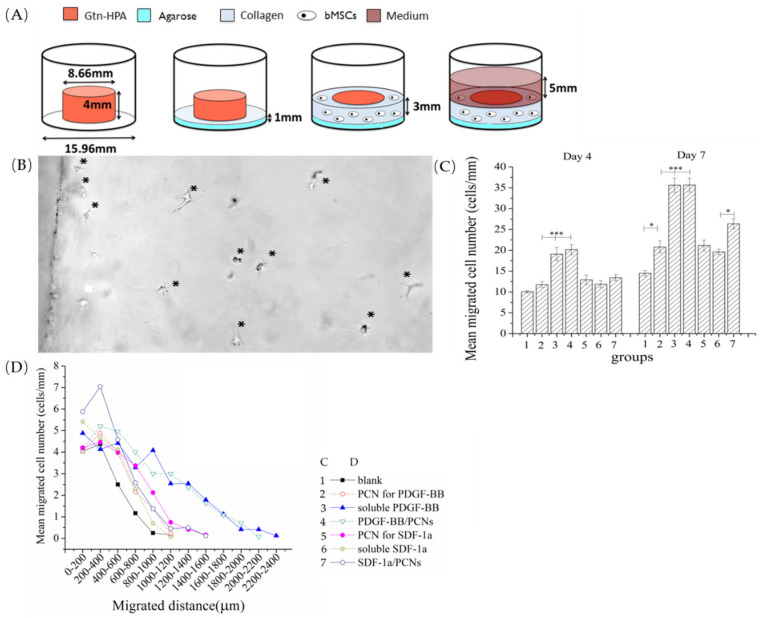
(**A**) Annulus-core migration model. (**B**) A typical migration micrograph on day 4; asterisks show bMSCs. (**C**) Migrated cell numbers/mm of interface in each group. (**D**) Migration distance on day 7 (* *p* < 0.05; *** *p* < 0.001).

Gtn-HPA gel was highly permissive of bMSC migration (*n* = 8). Under phase-contrast microscopy, by 4 days post-culture, cells were seen crossing the core-annulus interface and traveling a short distance into the blank Gtn-HPA cores (i.e., matrices with no growth factor incorporation; Figure 5B). There was approximately a 30% increase in the number of cells in the blank matrices from 4 to 7 days, with most cells remaining within 500 µm from the interface (Figure 5C,D).

The incorporation of 2 µg/mL soluble PDGF-BB directly into the gel resulted in a 2-fold increase in the number of cells found in the gel after 4 days, compared to the blank control (Figure 5C). This number of cells then again doubled from 4 to 7 days. After 7 days, the number of cells in the PDGF-BB-containing matrices (30 ± 1.6 cells/mm) was almost 3-fold greater than in the blank control (12.8 ± 0.9 cells/mm). At both 4 and 7 days, Gtn-HPA containing 2 µg/mL PDGF-BB encapsulated into PCNs displayed similar numbers of cells in the gels, compared to the gels with soluble PDGF-BB. Interestingly, adding PCNs alone (without PDGF-BB) resulted in a slight (~20%) increase in the number of cells in the gel compared to the blank control.

In contrast to the substantial effect that PDGF-BB had on increasing the number of bMSCs in the Gtn-HPA matrix, SDF-1α attracted fewer cells into the Gtn-HPA core gel both as soluble protein incorporated directly into the gel and when encapsulated first into PCNs for subsequent addition to the Gtn-HPA. The gels with the soluble SDF-1α had about 20% more cells than the blank controls after 4 and 7 days. While encapsulation of the SDF-1α into the PCNs had no notable effect on cell number after 4 days, after 7 days there was a greater number (~20% greater) of bMSCs in matrices containing SDF-1α/PCNs compared to matrices directly incorporating soluble SDF-1α. As found for the blank PCN control for the PDGF-BB/PCNs, the blank PCN control for the SDF-1α/PCNs demonstrated a greater number (%) of cells in the matrices compared to the blank matrix alone (Figure 5C).

Two-factor ANOVA demonstrated significant effects of group (*p* < 0.0001; power = 1) and time (*p* < 0.0001; power = 1) on the number of cells found within the matrices. Fisher’s PLSD post hoc testing demonstrated statistically significant differences for all group comparisons except for the following: blank PCN control for the PDGF-BB versus blank PCN control for the SDF-1α; blank PCN control for the PDGF-BB versus soluble SDF-1α; soluble PDGF-BB versus PDGF-BB/PCNs; and blank PCN control for SDF-1α versus soluble SDF-1α.

For the data obtained at day 7, 1-factor ANOVA followed by Fisher’s post hoc testing demonstrated the number of cells in the matrices containing SDF-1α/PCNs was significantly greater than that with the blank PCNs, and the number of cells in the matrices containing soluble SDF-1α was greater than that in the blank gel control group.

In addition, of interest was the longer distance inside the matrix traveled by the bMSCs under the influence of PDGF-BB (Figure 5D). On day 7, in both the soluble PDGF-BB group and the PCN-encapsulated PDGF-BB group, bMSCs were found as deep as 2 mm into the matrix. In comparison, in the blank matrices (with no growth factor or PCN), cells were found principally within 0.1 mm of the interface with the cell depot. For the SDF-1α groups, the encapsulated SDF-1α attracted bMSCs deeper into the matrix than the soluble SDF-1α group. However, of note was that cells migrated farther into the matrices containing blank PCNs (at the dose used for the SDF-1α/PCN group) than into the groups with soluble SDF-1α and SDF-1α/PCNs.

### 3.4. Proliferation of bMSCs in Gtn-HPA Matrices

**Figure 6:**
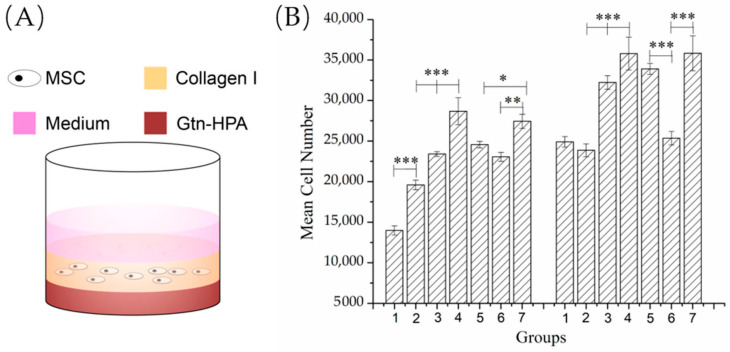
(**A**) Bilayer proliferation model. (**B**) Proliferated cell numbers on days 4 and 7. Groups are (1). Blank; (2) PCN for PDGF-BB; (3) soluble PDGF-BB; (4) PDGF-BB/PCNs; (5) PCNs for SDF-1α; (6) soluble SDF-1α; (7) SDF-1α/PCNs (* *p* < 0.05; ** *p* < 0.01; *** *p* < 0.001).

The fact that the number of cells appearing in the matrices under the influence of the MSC chemoattractants, PDGF-BB and SDF-1α, likely reflected some degree of proliferation and migration from the tissue-simulating cell-seeded annular construct, prompted the separate analysis of the effects of the growth factors released from the Gtn-HPA on bMSC proliferation in the collagen gel serving as a tissue simulant (Figure 6A).

By 4 days, there were substantial increases (almost 2-fold or greater) in the bMSC numbers in the collagen gel induced by the two growth factors whether in a soluble form added directly to the matrices or delivered by the PCNs: an increase from 13,979 ± 562 cells, for the blank matrix control to 23,403 ± 307 cells for the PDGF-BB, 28,673 ± 1675 cells for PDGF-BB/PCNs, and 27,433 ± 873 cells for SDF-1α/PCNs (*n* = 6; Figure 6B Day 4). The highest cell numbers were recorded in the matrices containing the PDGF-BB/PCNs and SDF-1α/PCNs. There were increases in the number of cells in all matrix groups from 4 to 7 days. At 7 days, same as at 4 days, the highest cell numbers were recorded in the matrices containing the PDGF-BB/PCNs and SDF-1α/PCNs. However, the increase in the number of cells in these groups (35,806 ± 2031 and 35,829 ± 2154 cells, respectively), above the blank control value (24,904 ± 641 cells), was approximately 40% instead of the near 2-fold increase registered at 4 days. At this time, the bMSC number increased more for the groups when the growth factors were delivered by the PCNs compared to the groups in which the soluble factors were added directly to the matrices (*n* = 6; Figure 6B Day 7). The soluble SDF-1α group, at 7 days, was similar to the control value with respect to the number of bMSCs in the matrix.

Of note was that the PCNs alone stimulated proliferation of the bMSCs in the collagen gel. At 4 days, there was an increase in the number of cells with an increase in the number of PCNs; proceeding from no PCNs to the dose of PCNs used for the PDGF-BB and then the higher dose used for the SDF-1α (Figure 6B). By 7 days, however, only the higher dose of blank PCNs was found to result in a higher cell number, compared to the blank control matrix.

Two-factor ANOVA demonstrated statistically significant effects of group (*p* < 0.0001; power = 1) and time (*p* < 0.0001; power = 1) on the number of bMSCs in the Gtn-HPA matrices. Fisher’s PLSD post hoc testing revealed statistically significant differences for all of the group comparisons except for PDGF-BB/PCNs versus SDF-1α/PCNs.

### 3.5. bMSCs Migration and Proliferation Response to PDGF-BB Dose

**Figure 7:**
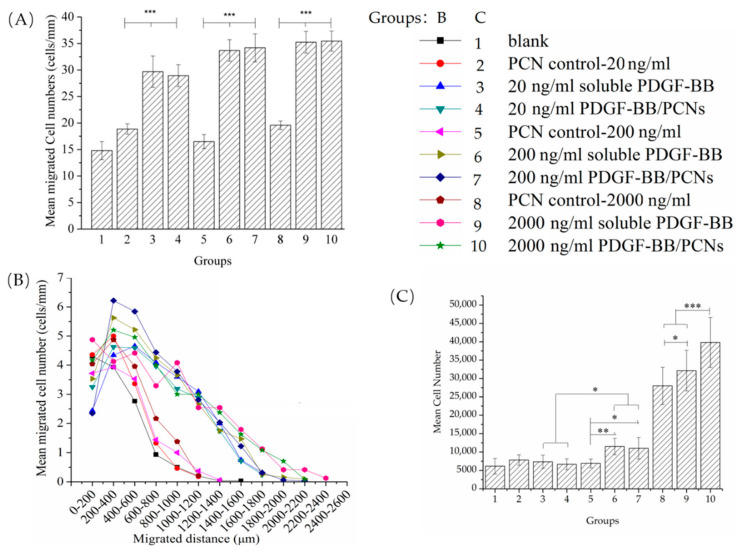
(**A**) Mean migrated cell numbers/mm of interface under the attraction of 20, 200 and 2000 ng/mL PDGF-BB, directly in gel or in PCNs; (**B**) Migration distance under different doses of PDGF-BB. (*n* = 6~8; *** *p* < 0.001). (**C**) Cell numbers after 7 days culture with different doses of PDGF-BB. (*n* = 6, * *p* < 0.05, ** *p* < 0.01, *** *p* < 0.001).

To further investigate the dose-response of bMSCs to PDGF-BB, increasing concentrations of PDGF-BB (20 ng/mL, 200 ng/mL and 2 µg/mL) were incorporated into the Gtn-HPA gel directly or into the PCNs. After 7 days, the lowest dose of PDGF-BB (20 ng/mL) added directly to the Gtn-HPA matrix or encapsulated into the PCNs resulted in a 2-fold increase in the number of cells found in the matrix, in the migration assay (Figure 7A): 30 ± 2 cells for both the soluble PDGF-BB group and the PDGF-BB/PCN group compared to 15 ± 1 cells for the blank PCN control group. An increase of the PDGF-BB from 20 to 200 ng/mL resulted in only a modest further increase (~10%) in the cell number in the matrices, to 34 ± 2 cells for both the soluble PDGF-BB group and the PDGF-BB/PCN group. There was no notable increase in the number of bMSCs in the matrices proceeding from 200 ng/mL to 2 µg/mL. At each of the 3 PDGF-BB doses, there was no difference in the cell numbers in the groups with the soluble PDGF-BB added directly to the matrix and with the PDGF-BB/PCNs. Addition of the blank PCNs to the Gtn-HPA as the respective doses resulted in no significant difference in the cell number in the matrices.

One-factor ANOVA showed a statistically significant effect of group on the number of cells in the matrices (*p* < 0.0001; power = 1). Fisher’s PLSD post hoc testing showed there were no statistically significant differences between the groups with the matrices containing the soluble PDGF-BB and the PDGF-BB/PCNs for all three PDGF-BB concentrations. The 3 PDGF-BB/PCN groups displayed statistically significant differences from their PCN controls. One-factor ANOVA of the blank matrix and 3 PCN control groups with increasing dose of PCN, as controls for the 3 respective PDGF-BB/PCN groups, showed there was no statistically significant effect of PCNs on the number of cells in the matrices (*p* = 0.06; power = 0.59).

Evaluation of the migration distance traveled by the bMSCs in the matrices after 7 days paralleled the results of the cell numbers (Figure 7B). The distances traveled by the cells in the 6 PDGF-BB groups were comparable, with cells reaching about 2 mm into the matrices, even for the lowest dose of PDGF-BB (20 ng/mL). In contrast, the bMSCs in the blank matrix and PCN control groups displayed similar migration profiles with cells only reaching about 1 mm into the matrices.

After 7 days, the low (20 ng/mL) and medium (200 ng/mL) doses of PDGF-BB showed little stimulus of the proliferation of the bMSCs whether in soluble form or encapsulated in the PCNs (Figure 7C). There was, however, a dramatic increase in the cell number (from 4- to 5-fold) in the 2 groups with 2 µg/mL of PDGF-BB, in soluble form and in PCNs, compared to the blank control. Additionally noteworthy was the increased number of cells in the group with the higher dose of PCNs. One-factor ANOVA revealed the statistically significant effect of group on the number of cells (*p* < 0.0001; power = 1). Fisher’s PLSD post hoc testing showed there were no statistically significant differences in cell number when comparing the 20 and 200 ng/mL PDGF groups with their respective control groups. All group comparisons among the two 2 µg/mL PDGF-BB groups and the PCN control group were statistically significant.

### 3.6. bMSC Auto-Osteogenic Differentiation in Gtn-HPA Gels

**Figure 8:**
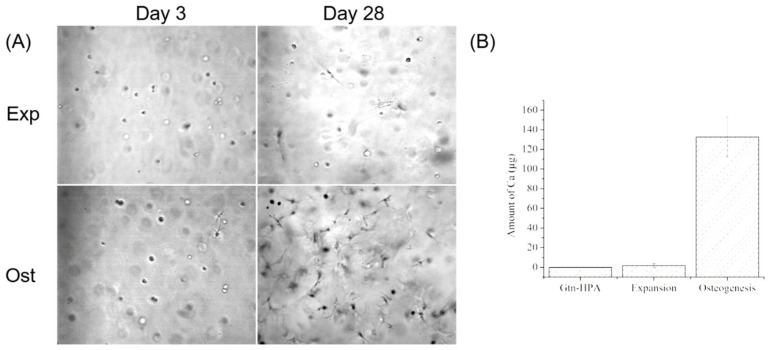
bMSC osteogenic differentiation and calcium deposition in 2% Gtn-HPA gels. (**A**) Morphology of cells in expansion medium and osteogenic medium on day 3 and day 28; (**B**) Amount of Ca mineralized by the cells in Gtn-HPA gels, quantified by ICP-AES.

During the 28 days culture, most of the cells in the expansion medium without FGF-2 kept their rounded shape while cells in osteogenic medium gradually spread out and accumulated to form nodules in gels (Figure 8A). After 28 days of culture, the gels in the expansion group were still homogeneous and transparent, whereas there were megascopic nodules formed in the osteogenesis group. The trace analysis of calcium measured by ICP-AES demonstrated there was almost no calcium mineralization (1.77 ± 1.77 µg) in the expansion environment compared to the cell-free gel which had no calcium, but differed significantly from the osteogenic group with 132.55 ± 20.41 µg of calcium (Figure 8B; *n* = 4; *p* < 0.0001; power = 0.9999). These results indicated that the bMSCs migrated into Gtn-HPA gels would host their stemness and avoid undergoing auto-osteogenic differentiation in general expansion medium, but they were easily induced to osteoblast and mineralized the calcium in their modified gelatin gel matrix.

## 4. Discussion

The notable findings of this study are: (1) the permissiveness of an injectable formulation of gelatin, capable of undergoing covalent cross-linking in vivo, of bMSC migration and proliferation; and (2) the enhancement of migration and proliferation of bMSCs in the gelatin matrix under the influence of PDGF-BB and SDF-1α released by PCNs. Of further note is the effect of PDGF-BB, which could be produced by the endothelial cells in vivo, in regulating the pericyte migration and vascular remodeling. Partial of the pericytes, known as mural cells, were identified as a subpopulation of MSCs [34].

An injectable natural biopolymer matrix capable of attracting endogenous MSCs into a provisional scaffold-filled defect and stimulating their proliferation could have many compelling applications. Prior studies have shown the tunable characteristics of the Gtn-HPA formulation [9,35]. Our own prior studies demonstrated the migration, proliferation, and differentiation of neural stem cells (NSCs) [10,16] and shockwave treated MSCs in Gtn-HPA [12]. It was also reported that the MSCs showed pro-angiogenic profiles via purely material-driven effects in Gtn-HPA. This gel directed both mouse and human bone marrow-derived MSCs to undergo endothelial differentiation through integrin (specifically α_1_ and α_v_β_3_ types)-mediated interactions at the cell-gel interface [14].

Compared with another gelatin-based biomaterial, gelatin-methacrylate (Gel-MA), which is a visible-light photopolymerizable hydrogel and has also been testified as permissible for MSC osteogenic and endothelial differentiation [36,37], Gtn-HPA has better tunable physicochemical properties, regarding the stiffness and gelation time. In addition, with the consideration of clinical applications, without using extra medical device for initiating gelation is also an advantage of Gtn-HPA. Although there is lack of applications when using Gtn-HPA as a 3D printing ink [38], it is theoretically potential to be adapted to extrusion-based, ink-jet and laser-assisted bioprinting but further material modification is required [39]. However, the 3D printable property is not necessary for many applications of repairing irregular-shaped defects.

Our prior work has demonstrated the utility of PCNs in delivering SDF-1α for the attraction of NSCs [16]; the current work has shown that PCNs are also effective in delivering PDGF-BB. While adding soluble growth factors directly to the Gtn-HPA matrix enhanced migration and proliferation of MSCs over controls, there were additional benefits seen in migration and proliferation in the growth factor/PCN groups in certain assays. This, coupled with the prolonged-release kinetics demonstrated in the current investigation and the proven bioactivity of the growth factors released by the PCNs, commend PCNs for future work for incorporation into the Gtn-HPA matrix.

SDF-1α did not attract as many bMSCs into the Gtn-HPA matrices as PDGF-BB. One explanation may be related to the fact that only a fraction of the adult goat bMSCs were found to stain for CXCR4, the main SDF-1α receptor. While those publications claim SDF-1 plays a critical role in murine MSC migration, the phenotype of the MSCs usually has CXCR4 stably expressed on the cell membrane [40]. Considering the role that SDF-1α may play in the chemoattraction of certain inflammatory cell types [41], PDGF-BB may be a more judicious selection for certain applications. Of interest in this study was that the low dose of PDGF-BB of 20 ng/mL was sufficient to attract most of the bMSCs into the matrix, compared to a dose of 2 µg/mL. Particularly of interest was that this low dose of PDGF-BB did not affect stimulating proliferation, indicating the increased number of bMSCs found in the PDGF-BB-containing matrices had migrated from the annular cell-seeded collagen gel.

One of the other interesting findings of the study is the enhancement of bMSC migration found with adding blank PCNs to the Gtn-HPA matrix. In the first migration experiment, after 7 days, PCNs alone (without PDGF-BB or SDF-1α) resulted in a slight increase in the number of cells in the gel compared to the blank control. In the second migration experiment, to assess the dose response to PDGF-BB, there was no effect of the PCNs alone on migration. Any positive effect of PCNs alone on migration may have been due to their binding of FGF-2, which may have diffused into the core matrix from the cell-seeded annular collagen gel construct. The PCNs may have adsorbed the FGF-2 and then gradually released it, thus enhancing the chemoattraction of the bMSCs and/or proliferation. As regards their effects in proliferation, at 4 days but not at 7 days, PCNs resulted in a more than 30% increase in cell number. In the second proliferation experiment, the higher dose of PCNs used as a control for the 2 µg/mL PDGF-BB/PCNs resulted in a substantially higher cell number after 7 days. Because FGF-2-free medium was used for the proliferation assay, the explanation provided above would not apply, and the explanation will require further study.

## 5. Conclusions

In conclusion, the Gtn-HPA matrix is highly permissive of bMSC migration. PDGF-BB incorporated directly into the gel or into PCNs greatly stimulates the number of migrating bMSCs and the distance they travel in the Gtn-HPA. PDGF-BB released from Gtn-HPA and from PCNs incorporated into the matrix also stimulates proliferation of bMSCs in an adjacent tissue-simulating (collagen) gel. The number of bMSCs that PDGF-BB recruited is dose-dependent, a low concentration of 20 ng/mL PDGF-BB could stimulate bMSCs migration into the target Gtn-HPA hydrogels effectively. However, due to the cytoplasmic arrangement of CXCR4 in bMSCs, SDF-1 α did not work as effectively as PDGF-BB in bMSC migration and proliferation, regardless of its encapsulation status. Despite there being no significant advantage by encapsulating PDGF-BB into PCNs on migration and proliferation assays taken on 3D Gtn-HPA and collagen hydrogel tissue simulating models in vitro, the Gtn-HPA hydrogels with PCNs encapsulated PDGF-BB still have the high potential application possibility to treat select defects. This is because the PCNs may provide the gel system a sustained release rate, and locate the growth factors in the defect area rather than diffusing into the surrounded normal tissue in a short time.

## Data Availability

Data available on request.
